# Dynamic observation and risk factors analysis of deep vein thrombosis after hip fracture

**DOI:** 10.1371/journal.pone.0304629

**Published:** 2024-06-03

**Authors:** Yuxuan Cong, Baohui Wang, Chen Fei, Hong Zhang, Zhong Li, Yangjun Zhu, Yan Zhuang, Pengfei Wang, Kun Zhang

**Affiliations:** 1 TCM Rehabilitation Department, Honghui Hospital, Xi’an Jiaotong University, Xi’an, Shaanxi Province, People’s Republic of China; 2 Pain Ward of Orthopedics Department of TCM, Honghui Hospital, Xi’an Jiaotong University, Xi’an, Shaanxi Province, People’s Republic of China; 3 Department of Orthopedic Trauma, Honghui Hospital, Xi’an Jiaotong University, Xi’an, Shaanxi Province, People’s Republic of China; 4 Department of Ultrasound, Honghui Hospital, Xi’an Jiaotong University, Xi’an, Shaanxi Province, People’s Republic of China; Istanbul University-Cerrahpasa, Cerrahpasa Medical Faculty, TURKEY

## Abstract

**Objective:**

To dynamically observe the occurrence of deep vein thrombosis (DVT) after a hip fracture and analyze of the risk factors affecting the dynamic alteration of DVT.

**Methods:**

Data of patients with hip fractures from January 1, 2017 to August 31, 2021 were collected. Patients were divided into DVT and non-DVT groups according to their daily Doppler ultrasonography findings. Survival analysis was used to describe dynamic changes in DVT occurrence with time. Log-rank tests were used to compare the influence of individual factors of patients with DVT occurrence, and a Cox proportional hazards regression model was used to identify the risk factors affecting the dynamic alteration of DVT occurrence.

**Results:**

A total of 331 patients were included: 148(44.7%) had preoperative DVT, and 143 (96.6%) had DVT in the first 3days after admission. The probability of DVT was 0.42 on Day 1, 0.11 on Day 2, 0.10 on Day 3, 0.08 on Day 4, 0.20 on Day 5, and 0.00 on Day 6–7, with a median survival time of 3.30 d. Age>70 years, intertrochanteric fracture, admission hemoglobin<130g/L, and admission hematocrit<40% had a significantly higher occurrence rate of DVT. A hematocrit level of <40% (Hazard Ratio 2.079, 95% Confidence Interval:1.148–3.764, P = 0.016) was an independent risk factor for DVT.

**Conclusion:**

DVT after hip fractures mainly occurred in the first three days after admission, the trend was stabilized within one week, and day 1 had the highest rate of DVT incidence. Age, fracture type, HGB level, and Hct level affected dynamic occurrence of DVT. At constant other factors, Hct<40% was 2.079-fold incidence in the risk of preoperative DVT formation than those with Hct≥40% after hip fracture.

## Introduction

Hip fractures (HF), typical osteoporotic fractures caused by low-energy fall injuries, commonly occur in older patients. Deep vein thrombosis (DVT) can occur any time after HF, and the hypercoagulability of blood increases the risk of DVT. Previous studies have shown that the incidence of preoperative DVT in patients with HF was between 6–62% [1–3]. Additionally, the high prevalence of multiple comorbidities makes older patients more susceptible to DVT than younger patients [4]. DVT is a predominant cause of pulmonary embolism (PE), a major cause of death after trauma [5]. Therefore, identifying the temporal regularity of DVT incidence and predicting its occurrence is essential in clinical management.

Previous studies have reported risk factors for DVT in patients with HP or lower extremity fractures, such as older age, prolonged preoperative time, fracture-related factors, and blood biomarkers [6–10]. However, these studies have mainly used static observation methods and lacked of dynamic observations of DVT formation, including timing of occurrence, location patterns, and dynamics of changes. The specific mechanism of DVT development over time requires further investigation. The primary aim of this study was to investigate the daily occurrence and dynamic alteration of preoperative DVT in patients with HF. The secondary aim was to analyze the hazard ratios of the associated factors and identify the risk factors to provide a reference for the prevention and therapy of preoperative DVT.

## Methods

### Ethics statement

This study analyzed the data of patients with HF admitted to the Honghui Hospital of Xi’an Jiaotong University from January 1, 2017, to August 31, 2021. This study was approved by the Ethics Committee of Xi’an Jiaotong University. All patients and minors’ parents provided written informed consent.

### Inclusion and exclusion criteria

**Fig 1:**
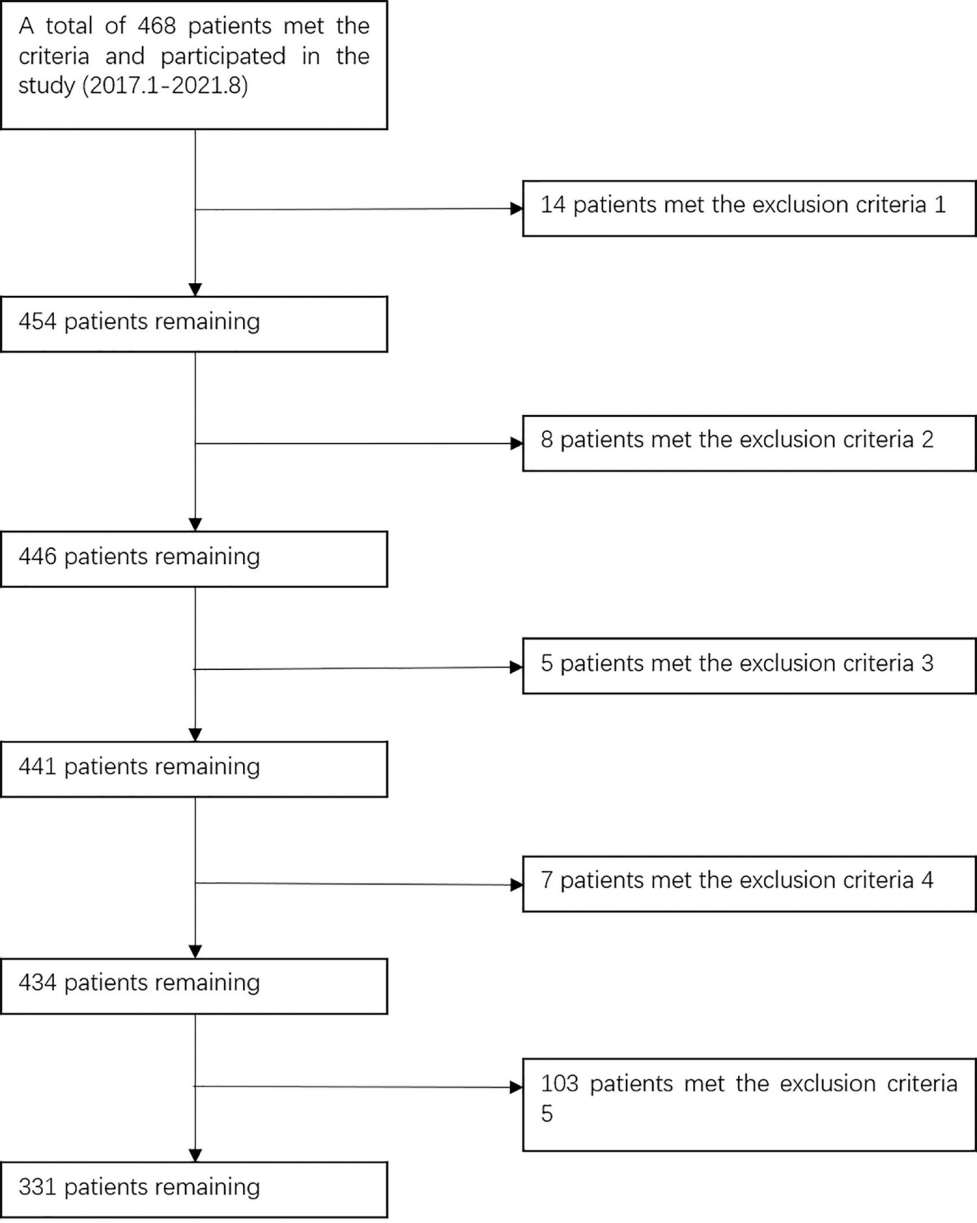
Derivation of the patient cohort.

Inclusion criteria: ①HF (including femoral neck fracture and intertrochanteric fracture); ② time from injury to admission <24h; ③ age ≥ 16 years; ④ unilateral closed HF; ⑤ patients who signed an informed consent form, who were able to receive anticoagulation therapy and could complete a preoperative ultrasound and other relevant examinations. Exclusion criteria: ① history of surgery in the lower extremity for any reason;②patients requiring anticoagulation therapy before the injury; patients contraindicated for anticoagulation; ③history of thromboembolic events;④pathological fracture, concurrent fracture in the lower extremity;⑤unable to complete examinations and incomplete medical records. The sampling procedure for all patients with HF is shown in Fig 1.

### Treatment

All patients admitted to the hospital, received low-molecular-weight heparin (LMWH) (0.4 mL, once a day; Glaxo Wellcome Production, GlaxoSmithKline, Tianjin, China) subcutaneous injection to prevent DVT. The LMWH dose was adjusted based on creatinine clearance [11]. In addition, mechanical thromboprophylaxis was administered (plantar venous pressure pump, 20 min, twice daily).

Color Doppler ultrasonography was performed to screen for DVT. The day of admission was defined as day 0; the first ultrasonography was performed on day 1 and then taken once per day until the day before surgery or day 7 (if the surgery was performed within 24h, ultrasonography was performed once before surgery). Experienced ultrasonographic radiologists blinded to the laboratory results scanned lower extremities. The diagnostic criteria were in accordance with those of the Robinov group’s criteria [12], which were confirmed based on the detection of venous lumen obstruction or filling defects. DVT cases were classified into three types: central (popliteal, femoral, and iliac veins), peripheral (calf muscle, fibular, and anterior/posterior tibial veins), and mixed (both central and peripheral) thromboses.

Patients with DVT received subcutaneous injections of LMWH (0.4 mL, twice daily), and were no longer taken Color Doppler ultrasonography. When preoperative ultrasonography results showed central or mixed thrombosis, an inferior vena cava filter was used if needed to prevent fatal pulmonary embolism. Anticoagulant therapy was discontinued 12h before surgery and resumed 24h after surgery. General information, DVT diagnostic results, and laboratory test data were collected from all the patients. Blood samples were collected on admission (within 24h of admission). Hematological indices included hemoglobin (HGB), hematocrit (Hct), D-dimer, alanine transaminase (ALT), aspartate transaminase (AST), prothrombin time (PT), activated partial thromboplastin time (APTT), and fibrinogen (Fg) levels.

### Statistics analyses

Statistical analyses were performed using SPSS (Version 19.0; SPSS Inc. Chicago, IL, USA). Continuous variables were assessed for normality with the Kolmogorov–Smirnov test, expressed with mean (± SD) or median (interquartile range) as appropriate. The statistical significance of continuous variables was examined using Student’s t-test or the Mann-Whitney U test, as appropriate. Categorical variables were evaluated using the chi-square test or Fisher ‘s exact test as appropriate. This difference was statistically significant (P<0.05).

We used survival analysis to describe the dynamic changes in DVT daily. We defined HF as initial event, DVT positivity as the outcome event, surgery patients as the censored data, DVT occurrence as patient developed DVT, survival time as the time interval from HF to DVT, and occurrence rate as the probability of patient developed DVT within time. The life table method was used to estimate occurrence rates and survival time. The log-rank test was used to compare occurrence rates between groups with different categorical variables. Survival curves were constructed using the life table and Kaplan-Meier methods. Before entering the Cox proportional hazard regression model, continuous variables were converted into categorical variables according to the cutoff values. All categorical variables (P < 0.05) were entered into the multivariate Cox proportional hazard regression model to identify independent predictors of DVT, and the correlation strength was indicated by the hazard ratio (HR) and 95% confidence interval (95% CI). A P-values of < 0.05 indicated statistical significance.

## Results

### Patient demographics and clinical characteristics

A total of 331 patients who met the inclusion criteria between January 1, 2017, and August 31, 2021, were included in the study. Based on ultrasonography results, patients were divided into DVT and Non DVT groups. The patient demographics are presented in Table 1.

**Table 1:** Patient characteristics according to preoperative ultrasound.

	Over all	DVT	Non DVT	P value
Number	331	148	183	
Age	70.58±14.74	74.70±11.68	67.26±16.09	0.000
Sex				
Male	124	46	78	
Female	207	102	105	0.031
Fracture side				
Left low limb	170	79	91	
Right low limb	161	69	92	0.509
Type of fracture				
Femoral neck fracture	168	64	104	
Intertrochanteric fracture	163	84	79	0.014
Medical morbidity				
Coronary heart disease(%)	91	46	45	0.188
Hypertension(%)	128	58	70	0.862
Diabetes(%)	55	28	27	0.311
Stroke(%)	55	30	25	0.108
Lung injury(%)	17	6	11	0.423
Associate trauma(%)	12	6	6	0.707
TFATS(days)	3.49±2.05	3.91±2.35	3.15±1.71	0.001
ASA classification				
0	97	41	56	
1	22	9	13	
2	154	66	88	
3	54	30	24	
4	2	1	1	0.499
Serum markers				
HGB (g/L)	120.90±18.39	117.01±16.93	124.06±18.95	0.001
Hct (%)	36.45±5.29	35.19±4.90	37.47±5.40	0.000
D-dimer (mg/L)	10.15±16.58	9.90±15.86	10.35±17.19	0.809
PT(s)	12.72±1.71	12.53±1.10	12.88±2.06	0.065
APTT(%)	35.76±20.86	33.68±18.98	37.45±22.18	0.109
Fg(g/L)	7.16±13.67	5.77±9.37	8.29±16.30	0.101
ALT(U/L)	18.07±8.95	17.07±7.67	18.94±9.87	0.105
AST(U/L)	21.09±7.20	21.60±7.00	20.65±7.37	0.306

Abbreviations: TFATS: time from admission to surgery; HGB, hemoglobin; Hct, hematocrit; PT, prothrombin time; APTT, activated partial thromboplastin time; Fg, fibrinogen levels; ALT, alanine transaminase; AST, aspartate transaminase.

**Fig 2:**
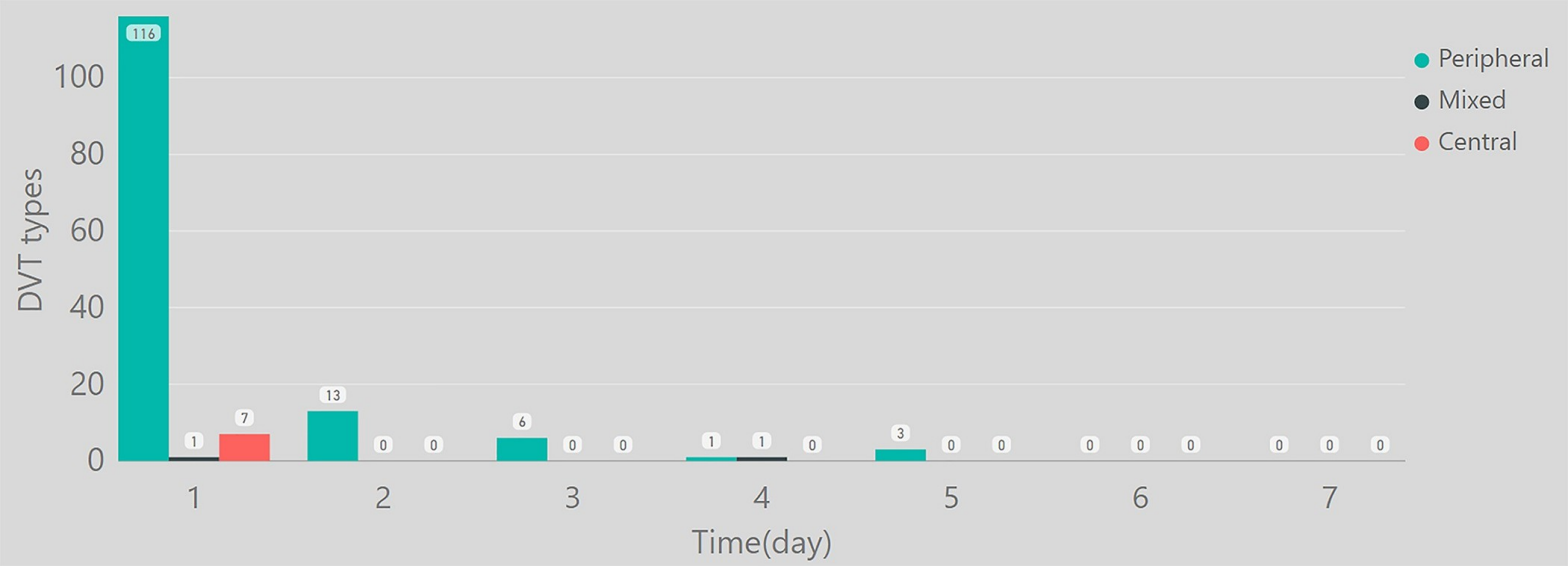
Daily incidence of DVT.

**Fig 3:**
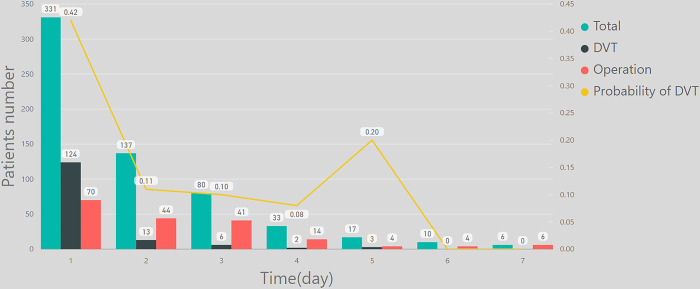
The probability of DVT on each day in survival analysis.

**Fig 4:**
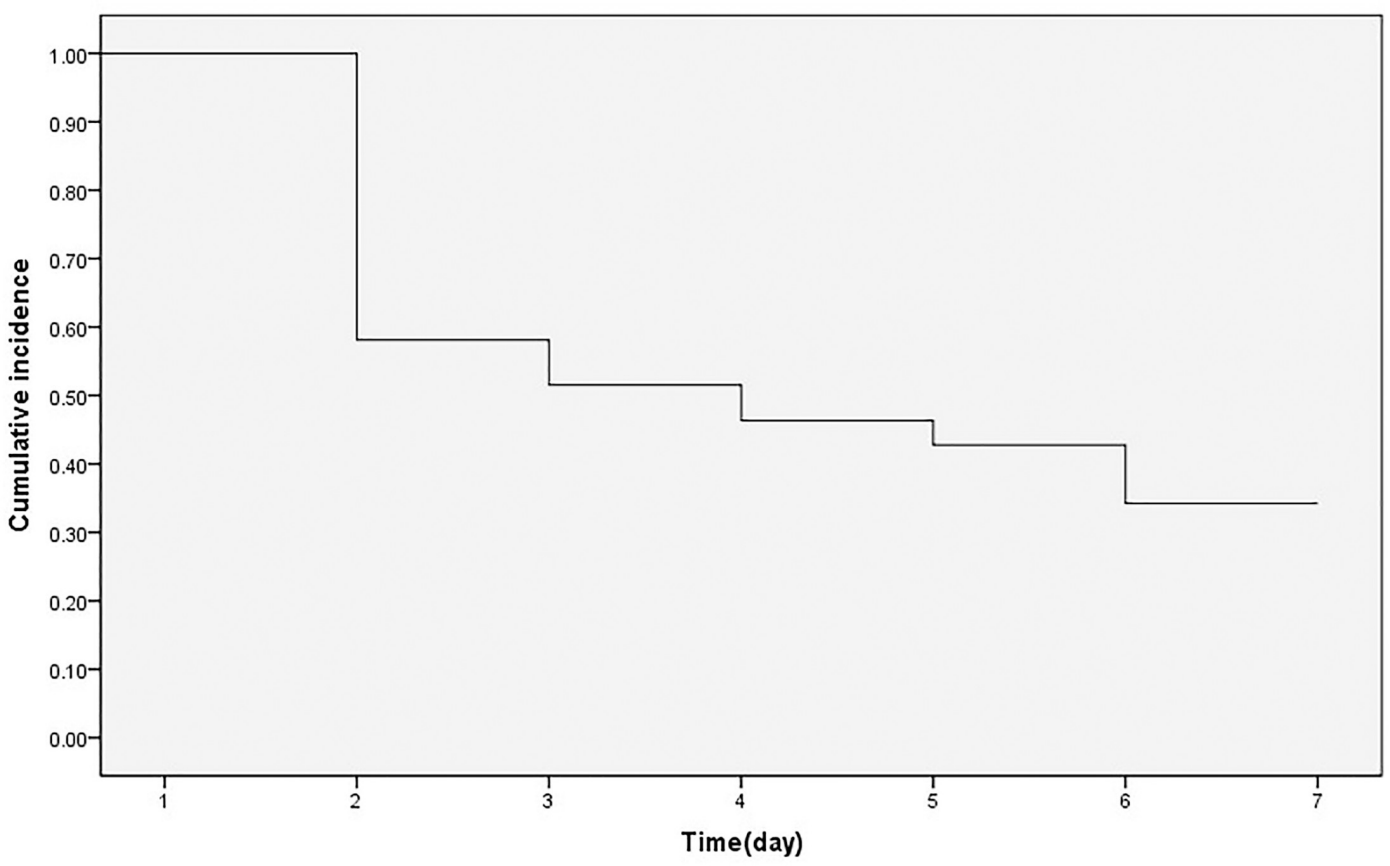
The survival curve of DVT in survival analysis.

Eleven patients received conservative treatment and 320 patients received surgical treatment. A total of 148(44.7%) patients were diagnosed with DVT. Of these 148 patients, 124 cases of DVT occurred on days 1–2, 13 on days 2–3, and 6 on days 3–4 (Fig 2), with 96.6% of the total DVT cases occurring in the first 3 days. In survival analysis, the probability of DVT was 0.42 on Day 1, 0.11 on Day 2, 0.10 on Day 3, 0.08 on Day 4, 0.20 on Day 5, and 0.00 on Day 6–7, with the median survival time of 3.30 d (Fig 3). The cumulative probability without DVT was: 0.58 on Day 1, 0.52 on Day 2, 0.46 on Day 3, 0.43 on Day 4, and 0.34 on Day 5-7(Fig 4).

A total of 139(93.9%) patients had peripheral DVT, seven (4.7%) had central DVT, and two (1.4%) had mixed DVT. A total of 54 patients developed DVT in the uninjured limb, and 23 developed DVT in bilateral limbs. Seven patients had preoperative inferior vena cava filters. None of the patients developed pulmonary embolism.

### Risk factors for DVT

A comparison of patients with and without DVT revealed statistically significant differences in sex and fracture type. The mean age of the DVT group was 74.70 ± 11.68 years (ranging from 17 to 94 years), and the mean time from injury to surgery was 3.91± 2.35 days, significantly higher than in the group without DVT. The mean admission HGB and Hct of the DVT group were 117.01± 16.93g/L and 35.19± 4.90% respectively significantly lower than in the group without DVT.

**Fig 5:**
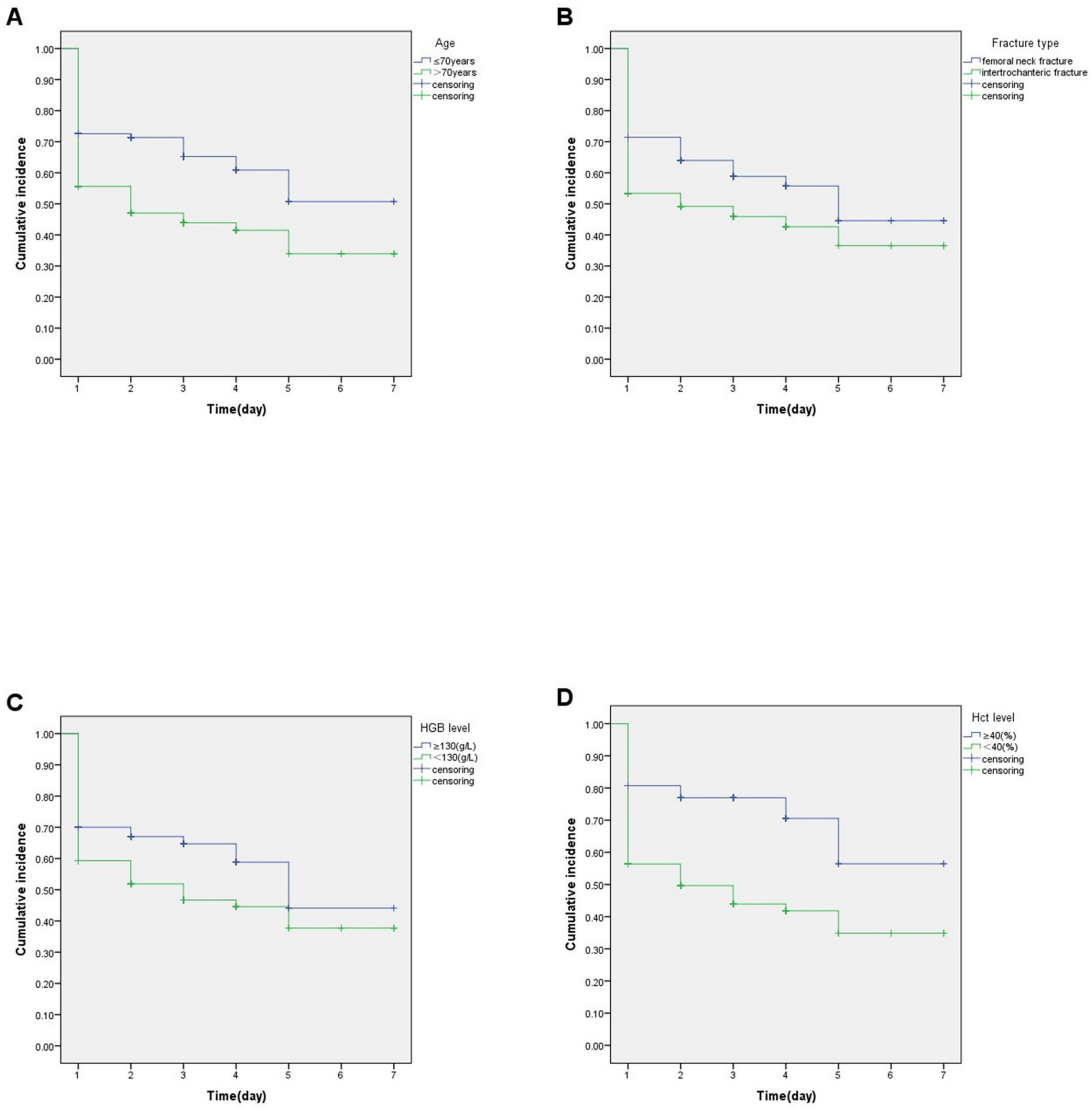
Kaplan-Meier curves for DVT occurrence with associated risk factors. Log-rank test for P-values. A: Cumulative incidence on gender age(P<0.05). B: Cumulative incidence on fracture type(P<0.05). C: Cumulative incidence on HGB level(P<0.05). D: Cumulative incidence on Hct level(P<0.05). Abbreviation: HGB, hemoglobin; Hct, hematocrit.

In the log-rank test, there was no statistical difference in the effect of sex on the occurrence rate. Patients had a lower occurrence rate of DVT with age ≤70 years (age ≤70 vs >70, mean survival time:4.72d, 95%CI:4.09–5.34 vs 3.56d, 95%CI:3.11–4.01, P = 0.000), femoral neck fracture (femoral neck fracture vs intertrochanteric fracture, mean survival time: 4.39d, 95%CI:3.87–4.91 vs 3.64d, 95%CI:3.12–4.16, P = 0.006), normal HGB level(HGB ≥130 vs <130, mean survival time: 4.49d, 95%CI:3.37–5.25 vs 3.78d, 95%CI:3.35–4.21, P = 0.022), and normal Hct level(Hct≥40% vs<40%, mean survival time: 5.18d, 95%CI:4.42–5.95 vs 3.61d, 95%CI:3.20–4.03, P = 0.000)(Fig 5).

### Impact on DVT occurrence

In the univariate analysis, age>70 years, intertrochanteric fracture, and admission Hct<40(%) were associated with a higher risk of DVT. In the multivariate analysis, Hct<40% (HR 2.079, 95% CI:1.148–3.764, P = 0.016) was an independent predictor and remained a statistically significant risk variable for DVT development(Table 2).

**Table 2:** Risk factors of DVT for hip fracture patient.

	Univariate analysis		Multivariate analysis	
	HR (95% CI)	p-Value	HR (95% CI)	p-Value
Age				
≤70	1			
>70	1.702 (1.193,2.427)	0.003	1.270(0.748,2.156)	0.375
Fracture type				
Femoral neck fracture	1			
Intertrochanteric fracture	1.457 (1.051,2.019)	0.024	1.179(0.797,1.743)	0.410
Admission HGB				
≥130 (g/L)	1			
<130 (g/L)	1.438 (0.983,2.103)	0.061		
Admission Hct				
≥40 (%)	1			
<40 (%)	2.291 (1.429,3.674)	0.001	2.079(1.148,3.764)	0.016

Abbreviations: HGB, hemoglobin; Hct, hematocrit.

## Discussion

To best of our knowledge, this is the first study to investigate DVT formation using daily Doppler ultrasonography, and explore the occurrence of DVT over time through survival analysis. Our primary findings were as follows: (a) 148/331 (44.7%) patients were diagnosed with DVT preoperatively, and 93.9% of DVT were peripheral. (b)DVT mainly occurred in the first 3 days after admission, especially from admission to day 1, and the trend of DVT formation stabilized from day 5 onward. (c) Age, fracture type, HGB, and Hct levels, affected the dynamic occurrence of preoperative DVT. (d) Hct level was an independent predictor of DVT survival. With other factors being constant, patients with Hct<40% had a 2.079-fold incidence in the risk of preoperative DVT than those with Hct≥40%.

Because the heightened risk of DVT begins immediately after a hip fracture, the period of thrombosis formation is relatively short, and blood clot formation can occur at any time, especially in older patients. Zuo [13] reported that 20.1% of 578 intertrochanteric fracture had DVT upon admission. Xing [4] reported a higher prevalence(29.8%) of DVT following hip fracture in older patients at admission. Of the patients with intertrochanteric fracture, 39.1% had preoperative DVT [14]. In Roberts’ study, this ratio increased to as high as 62% in HF when the time to surgery was delayed by more than 2 days [15]. In this study, some patients converted from a negative to a positive result owing to daily ultrasonography, resulting in a higher incidence rate (44.7%) than in those with only one ultrasonography. We also found that 96.6% of DVT cases occurred in the first 3 days, and the probability of DVT occurrence on day 1 was the highest at 0.42, which demonstrating that DVT mainly occurred in the early post-injury period. This could be caused by the hypercoagulability of blood with time and the body’s post-trauma stress, both at a dynamic peak within four days after trauma [16]. Furthermore, DVT might occur immediately after injury before ultrasonography, which explain the high incidence of DVT on day 1. Therefore, screening for DVT in patients with HF upon admission is necessary. Meanwhile, the prophylaxis after HF is critical, which could reduce the risk of DVT [17], and was recommended by major health organization [18]. In patients without therapeutic anticoagulation, a significant rate of patient acquired isolated distal DVT progression to proximal DVT was observed [19].

The probability without DVT in patients decreased with prolonged time after admission, from 1.00 to 0.34 on day 1 to day 5, and stabilized at 0.34 from day 5 to day 7. The mean daily probability of DVT occurrence was 0.13. The risk of DVT gradually increased with time after injury, and Zuo et al showed that every delay of 1 day from admission was associated with a 37% increased risk of DVT [13]. Even with anticoagulation therapy, a longer time before surgery has been reported as a relevant factor [20, 21]. Our result was the same as that Day’s study [19], which showed that thromboembolic progression occurred within 7 days. Therefore, it reminded surgeons that repeated ultrasonography should be performed within 7 days for patients with longer preoperative time.

Previous studies have used logistic regression to analyze the effects of risk factors on DVT formation. In this study, we used survival analysis to observe the dynamic alteration in DVT incidence and compared the HRs of risk factors affecting DVT development with time. Yeol [22] reported the incidence of DVT after major lower limb orthopedic surgery and found a 5-fold increased risk in patients of 50–69 years and a 10-fold higher risk in those age > 70 years then in those aged < 49 years. Based on previous literature and the average age of the study sample, we chose 70 years as the cut off value. The result showed that the survival rate was significantly different in different age groups, and the HR was 1.702, indicating that the age>70 years group had a 1.70-foldgigher risk of DVT when considering age. An acquired prothrombotic state, decreased vascular function, and decreased renal function may increase DVT risk in older individuals [4, 8].

The DVT occurrence rate varied across HF types, intertrochanteric fractures had a higher occurrence rate, and had a 1.46-fold incidence in the risk compare with femoral neck fractures. McNamara et al [23] demonstrated that intertrochanteric fractures were a risk factor for symptomatic venous thrombus embolism(VTE), and incidence of intertrochanteric fracture was twice as high as that of intracapsular HF. Therefore, surgeons should note that intertrochanteric fractures are associated with a higher risk of DVT than femoral neck fractures perioperatively.

We considered that blood loss after HF, including hidden blood loss that was not easily detected, caused the initiation of the coagulation response, which was the main reason for DVT development in the early post-injury period. In this study, indicators of blood loss after fracture were associated with DVT formation. Patients with HGB levels of <130 g/L had a higher occurrence rate. In multivariate analysis, Hct was an independent risk factor of preoperative DVT, which meant that with other factors being constant, the patients with Hct<40% had a 2.079-fold incidence in the risk of DVT than those with Hct≥40%. Kumar reported a sustained downward trend in HGB after HF [24], and suggested potential anemia caused by persistent hemorrhage after HF. Wu et al [25] showed that the preoperative decrease in HGB was nearly 21.55 g/L in patients with extracapsular HF and nearly 15.63 g/L in patients with intracapsular HF The largest differences in HGB levels were observed on the first and second days after admission. Anemia is a common condition in older patients and has been demonstrated to increase the risk of DVT [26]. Blood loss is an important factor in promoting hypercoagulability [27], leading to the activation of the coagulation system and a higher level of hypercoagulability, which may contribute to the development of DVT [8]. Additionally, differences in blood loss may explain the different occurrence rates of different fracture types.

This study had a few limitations. First, incomplete data were eliminated, and the authenticity of the results must be evaluated. Second, although Color Doppler ultrasonography is not the gold standard for diagnosing DVT and the operating skills of different sonographers varied, our study was conducted by senior sonographers, which minimized the impact on outcomes. Third, although we limited the time between injury and admission, the first ultrasonography could not be performed immediately. However, on the day after admission, the trends in DVT formation during this time could not be specified. Finally, multi-center studies with larger samples are needed to confirm the validity of our results.

## Conclusion

DVT after HF mainly occurred in the first 3 days after admission; the trend stabilized within 1 week, and day 1 had the highest DVT incidence rate. Patients age>70, intertrochanteric fracture, admission HGB<130g/L, and admission Hct<40%, had a higher tendency to develop DVT. Hct level was an independent predictor (HR 2.079, 95% CI:1.148–3.764, P = 0.016) for the dynamic development of DVT after HF, which means that with other factors being constant, the patients with Hct<40% had a 2.079-fold incidence in the risk of preoperative DVT formation than those with Hct≥40%. Targeted precautionary measures should be implemented in the early post-injury period.

## Supporting information

S1 Checklist STROBE statement—checklist of items that should be included in reports of observational studies. (DOCX)

S2 Checklist TREND statement checklist. (DOCX)

S1 Data (XLSX)
